# Lift-Out Specimen Preparation and Multiscale Correlative Investigation of Li-Ion Battery Electrodes Using Focused Ion Beam-Secondary Ion Mass Spectrometry Platforms

**DOI:** 10.1021/acsami.4c12915

**Published:** 2024-10-09

**Authors:** Pablo Maria Delfino, Mariia Bofanova, Eric De Vito, Nicolas Dupré, Guillaume Lamblin, Willy Porcher, Tom Wirtz, Jean-Nicolas Audinot

**Affiliations:** † Advanced Instrumentation for Nano-Analytics (AINA), Luxembourg Institute of Science and Technology (LIST), L-4422 Belvaux, Luxembourg; ‡ University of Luxembourg, L-4365 Esch-sur-Alzette, Luxembourg; § University of Grenoble Alpes, CEA-Liten, 38000 Grenoble, France; ∥ Nantes Université, CNRS, Institut des Matériaux de Nantes Jean Rouxel, IMN, 44000 Nantes, France; ⊥ Transparent and Optically Tuneable Materials and Nanostructures, Luxembourg Institute of Science and Technology (LIST), L-4422 Belvaux, Luxembourg

**Keywords:** secondary ion mass spectrometry (SIMS), lithium-ion battery, focused ion beam (FIB), post-mortem analysis, lift-out, correlative microsopy

## Abstract

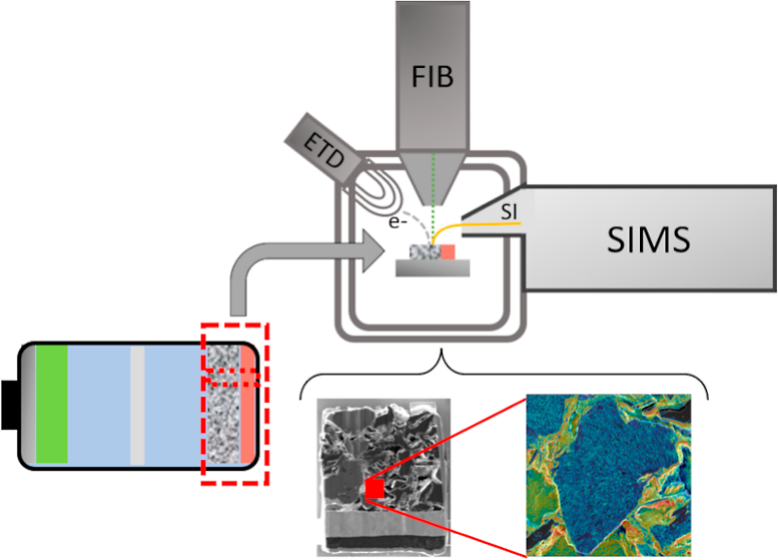

Advanced characterization is paramount to understanding battery cycling and degradation in greater detail. Herein, we present a novel methodology of battery electrode analysis, employing focused ion beam (FIB) secondary-ion mass spectrometry platforms coupled with a specific lift-out specimen preparation, allowing us to optimize analysis and prevent air contamination. Correlative microscopy, combining electron microscopy and chemical imaging of a liquid electrolyte Li-ion battery electrode, is performed over the entire electrode thickness down to subparticle domains. We observed a distinctive remnant lithiation among interparticles of the anode at the discharge state. Furthermore, chemical mapping reveals the nanometric architecture of advanced composite active materials with a lateral resolution of 16 nm and the presence of a solid electrolyte interface on the particle boundaries. We highlight the methodological advantages of studying interfaces and the ability to conduct high-performance chemical and morphological correlative analyses of battery materials and comment on their potential use in other fields.

## 1 Introduction

The global demand for energy storage in recent decades has boosted the development of alternative technologies to fossil fuels. In mobile applications, for example, particular interest has been paid to rechargeable lithium-ion batteries (LIBs) due to their superior performance compared to that of other battery technologies. To date, tremendous progress has been made in terms of increasing the energy density, extending the lifetime, and improving safety. However, with this progress comes a greater demand for improved characterization methodologies to better understand the physical, chemical, and electrochemical processes taking place during cycling. More specifically, detailed descriptions of the solid electrolyte interface (SEI) formation, lithium transport mechanism within cell components, loss of lithium and/or active materials, and their evolution during the battery life are pivotal in pinpointing the origin of battery performance loss.^1−4^

Among different analytical characterization techniques, secondary-ion mass spectrometry (SIMS) is particularly suitable for LIB applications as it can detect all elements and their isotopes. SIMS is especially sensitive to detect lithium, which has a high useful yield (Li ions detected/Li atoms sputtered).^5^ This makes SIMS an excellent technique for studying model electrochemical cells utilizing isotope-enriched components, for example, in lithium dynamics investigations.^6,7^ Moreover, SIMS as a surface-sensitive analysis technique is of special interest for interface investigations, for instance, SEI analysis. However, nowadays, SIMS chemical mapping is mainly limited by the spatial resolution and the data being prone to topographic artifacts. Indeed, the SIMS signal intensity is affected by the sputtering rate, which is dependent on the material and the incidence angle of the incoming ion, which introduces artifacts of visualization and interpretation.^8,9^ In situ SIMS analyses of LIBs (observations made exactly in place where the cycling is performed) give insights into more fundamental aspects of the structure and chemistry of the interfaces but face technical challenges that limit their use to specific cell designs only, excluding investigations of practical battery configurations.^10,11^ On the other hand, ex situ or post-mortem analyses (after the cell is disassembled) enable a large variety of cell components and materials to be surveyed in realistic cycling conditions. However, this approach faces a series of practical challenges to achieve valuable results.^9^ First, lithium and its compounds formed in the interfaces of the battery components are highly reactive to air and water. Hence, to preserve their chemical nature, it is paramount to protect the electrodes from contaminants from the cell opening (i.e., in an Ar glovebox) to the analysis chamber of the instrument. This aspect restricts the population of instruments for this application as it requires a dedicated airtight transfer system scarcely available in commercial instruments. Second, in the past few years, scientists in pursuit of materials with improved energy density and cycling performance have developed compounds with a nanometer-scale architecture. To untangle the (de)lithiation and degradation mechanisms of these new materials, characterization techniques with sufficiently high spatial resolution are required, thus limiting the number of SIMS instruments available on the market. Third, dedicated SIMS instruments typically do not provide topographic and structural data, hindering a direct correlation between chemical information and surface morphology or roughness. To address this issue, a correlative analysis approach is often necessary. Lastly, SIMS requires the samples to be prepared appropriately in order to obtain reliable results and take advantage of the instrument’s capabilities. To this end, the samples need to be ultrahigh-vacuum-compatible and preferably conductive and flat [ref (12) Ch. 15]. At least a partial remotion of liquid electrolytes is required, not only to obtain a good vacuum for the analysis but also to avoid solid residues after solvent evaporation, as these act as electrical insulators and obstruct access to the underlying material. The main pitfall in performing SIMS analyses on rough materials is that the roughness leads to preferential erosion. This is particularly true in battery characterization as the electrodes are composed of an agglomeration of microparticles with irregular shapes and rough finishing. In recent years, add-on SIMS systems integrated into focused ion beam (FIB) instruments have emerged as an accessible solution that combines structural, morphological, and chemical information in a single instrument.^13^ The high brightness provided by the ion source and suitable ion optics combined with high-transmission secondary ion (SI) mass spectrometers offers highly sensitive analytical information with high spatial resolution while preserving the original FIB-SEM instrument capabilities for analytics, electron imaging, and micromachining.^14,15^

In this study, we present a complete workflow for the correlative chemical/elemental and morphological characterization of LIB electrodes using FIB-SIMS platforms. Our results demonstrate the capability of these instruments to prepare the sample and study adequately the chemical/elemental composition and structure of the battery electrodes with nanoscale lateral resolution and high sensitivity, thereby addressing environmental contamination problems and mitigating artifacts resulting from the morphology. A lift-out specimen extraction approach with location selectivity over the electrode not only permits multiple scale domains to be studied, including the entire electrode thickness from the current collector to the electrode–electrolyte interface, but also enables a localized analysis of subparticle domains and interfaces. To the best of our knowledge, this is the first reported study of a battery lift-out specimen analysis that exploits elemental SIMS analysis with such a high spatial resolution. Moreover, the methodology presented here can be extended not only for studying LIB systems (both positive and negative electrodes) but also for correlative analytical/morphological investigations of multilayer structures, coatings, and metallic and composite materials.

## 2 Experimental Section

### 2.1 Cell Preparation

Half-cells were prepared with negative electrodes manufactured in-house and Li metal as the counter electrode. The negative electrode active material consisted of 64 wt % of graphite, 31 wt % of second-generation silicon–carbon composite (UM1300IE from Umicore),^16^ 2% of Na-CMC, 0.9% of carbon black, 0.1% of carbon nanotubes, and 2% of latex. A 10 μm thick bare copper foil was coated to achieve a targeted active material with a specific capacity of 648 mA h g^–1^ and an areal reversible capacity of 3 mA h/cm^2^. Subsequently, the electrodes were calendared at a thickness of 50 μm and a porosity of around 40%. Electrodes disks of 16 mm in diameter were cut and assembled with a separator and Li metal (Goodfellow, Li metal ribbon, 0.75 mm thickness, 99.9%) and filled with an electrolyte (Sigma-Aldrich, LiPF_6_ 1.0 M EC/EMC 1:1) by using El-Cell test cells. A BioLogic cycler BSC 805 at C/20 rate was used for a single charging and discharging with a potential cutoff of 0.01 and 1 V, respectively. The cells were carefully disassembled, rinsed gently with dimethyl carbonate, and mounted on a conventional 12 mm-diameter electron microscope stub using a double-sided carbon sticking tab. Both the assembly and disassembly of the cell were carried out inside an Ar-filled glovebox with *p*(H_2_O and O_2_)/*p* < 2 ppm. Subsequent sample transfers in and out of the FIB-SIMS platform were executed using airtight transfer shuttles and load-lock systems (Ferrovac AG and Semilab) under inert gas (i.e., high purity Argon) avoiding contact with the atmosphere throughout the entire transfer process. It is worth noting that these systems allow the venting of the load-lock chamber and shuttle interior with a chosen gas, i.e., inert gas, with the sample always being kept in an inert environment either for storage in the glovebox or for further analysis in a different instrument. We believe that this is the correct procedure to allow the material surface to remain unperturbed since a differential positive pressure in the shuttle interior prevents the ingress of air, thereby discouraging the use of static vacuum, which can rapidly expose the sample surface to an unprotected environment.

### 2.2 FIB-SIMS Platforms

For the present study, two different FIB-SIMS platforms, equipped with compact magnetic sector SIMS spectrometers designed in-house, were utilized. The spectrometer is an add-on attachment that can be installed in commercially available FIB platforms, offering high spatial resolution and a high-sensitivity analytical solution for chemical mapping including isotope selectivity. It consists of a retractable SI extraction box, transfer optics, magnetic sector, and an ion detection system. The retractability of the extraction box allows normal use with no detriment to the original instrument performance and enables correlative analysis. Among the different analytical tools,^17^ magnetic sector mass spectrometers offer parallel mass detection with high overall transmission, presenting high sensitivity and fast chemical imaging simultaneously.^14,15,18^ By applying a sample bias, either positive or negative SIs can be collected and analyzed, permitting the parallel detection of four different masses in the four channeltron configuration or the full mass spectrum (MS) with the continuous focal plane detector.^19^ Two FIB-SIMS systems were used in this work. A dual beam FIB-SEM Scios from Thermo Fisher Scientific was equipped with a ^69^Ga^+^ ion column and an electron field emission column for SEM operations. The Ga-FIB column can operate at acceleration energies from 500 eV to 30 keV and probe currents of sub-pA to 65 nA. Further information on the analytical performance can be found in ref (14). Moreover, it is equipped with a micromanipulator (Kleindiek MM3A) for lift-out procedures. The second FIB-SIMS platform is a NanoFab helium ion microscope (HIM) from Zeiss equipped with a high-brightness ion column that can operate with He^+^ or Ne^+^ ions. For SIMS operation, it offers a detectable mass range from 1 to 500 amu with a resolving power of 500 and sub-15 nm lateral resolution in image mode. For a more in-depth description of the HIM-SIMS instrument, readers can refer to ref 15. ImageJ 1.53t was used for image data treatment and plotting. Finally, a scientifically derived color map was implemented for the image visualization of SIMS data to respect scientific and inclusive data interpretation.^20^

## 3 Results and Discussion

### 3.1 Lift-Out Specimen Preparation

**Figure 1:**
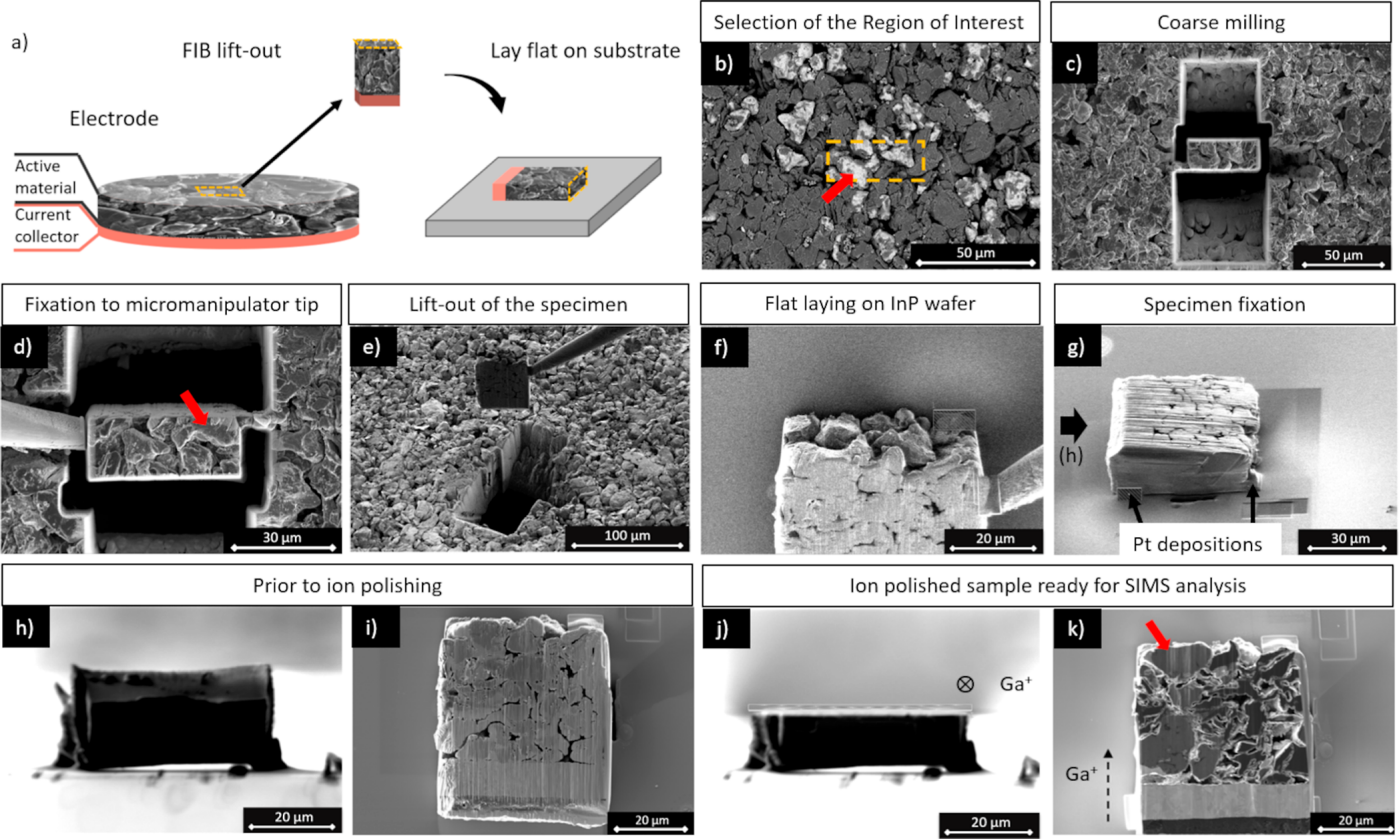
Lift-out sample preparation steps for SIMS analysis. (a) Schematics of the FIB lift-out positioning for SIMS analysis. (b) Backscatter electron image of the electrode with the selection of a ROI. The brighter particles correspond to the silicon–carbon composite and the darker to graphite. (c) Course milling around the ROI, leaving one connecting bridge on the left. (d,e) Lift-out with a micromanipulator. (f) Flat-laying specimen on an InP wafer and (g) fixation with ion-beam-induced platinum deposition. (h,i) Specimen as deposited with rough top finishing. (j,k) Specimen after ion polishing ready for SIMS analysis. The red arrow allows the tracking of one of the silicon–carbon composite particles.

The negative electrodes used for this investigation were self-manufactured and are composed of copper foil coated with electrochemically active material. The main constituents of the active material are graphite and second-generation silicon–carbon composite particles. The complete FIB preparation is carried out on a Ga FIB-SEM-SIMS tool equipped with all the accessories needed for a conventional lift-out workflow. Further information on the manufacturing of the electrodes and the description of the instruments used throughout this work can be found in Sections 2.1 and 2.2, respectively. After the sample was introduced into the instrument chamber, the desired region of interest (ROI) was selected. For this purpose, electron imaging by scanning the sample with the electron beam of the FIB-SIMS was favored as it can be considered nondestructive. The backscattered electron (BSE) signal provides density and atomic number contrast, facilitating a reliable way to distinguish different phases. Hence, the principal components of the anode active material were discerned as an agglomeration of the silicon–carbon composite and graphite particles (Figure 1b). This site-specific capability can be provided by other probing techniques available in these instruments; for instance, a preliminary chemical mapping by EDS or cathodoluminescence material contrast can aid in the selection of the ROI. In this stage, a protective layer can be applied by FIB-induced deposition (i.e., Pt or W), covering an area slightly larger than the ROI. It was not applied here as the preservation of the uppermost surface was not of primary importance for this investigation. Next, the surrounding material was removed by opening two opposite triangular-shaped trenches (with 60 × 60 μm^2^ and 50 × 50 μm^2^ square bases) and prismatic holes, leaving only a narrow connecting bridge (Figure 1c). The milling was applied gradually all around the specimen to avoid rejoinings by redeposition until the full thickness of the electrode had been penetrated. The stage was tilted to 45° with respect to the axis of the Ga-FIB to free the bottom of the specimen, and the lateral connection was reduced to a narrow part at the top side. This course milling process was executed using a Ga^+^ beam of 65 nA and 30 keV. The specimen was lifted out using a micromanipulator, positioned on a substrate (i.e., indium phosphate wafer) by laying it flat and fixed with ion beam-induced platinum deposition (Figure 1d–f). Up until this step, this preparation procedure can be considered an adaptation of the well-established TEM lift-out lamella preparation. Due to it containing Si as a component of the active material, an InP substrate was chosen for this example, rather than a Si wafer, to avoid having a foreign Si signal in the electrode material (e.g., by redeposition). The specimen was placed in such a way that it made full contact with the substrate and was attached carefully at several fixation points to ensure good mechanical stability (Figure 1g). To further reduce the roughness of the cross-sectioned surface (Figure 1h,i), ion polishing was performed. First, the stage was tilted so that the plane of the substrate was parallel to the axis of the Ga-FIB. Second, the sample was oriented so that the Ga ions impinge first on the copper current collector and the active material downstream, as shown in Figure 1j,k. Once the sample is correctly positioned, the material of the cross-section was gradually removed layer by layer applying ion beam current between 15 and 1 nA in descending order. Here, the compact and homogeneous layer of copper acts as a shielding plate enhancing the uniform and gradual removal of the active material, thereby reducing the extent of ripple formation or curtain effect.^21^ The final surface of the specimen is flat, smooth, and parallel to the substrate (Figure 1j), and the internal structure of the electrode is revealed as shown in Figure 1k. The site-specificity shown in Figure 1b is exemplified by the grain indicated with the red arrow observed in the cross-section (Figure 1k). Since the electrodes are dried before being investigated and low-conductive sample holders are used, there is no risk of lithium migration in the system. However, it should be noted that electrodes with active electrolytes (typically solid-state electrolytes) may induce potential variations within the system, requiring attention to a solution to keep samples away from any contact with the ground of the instrument.

### 3.2 SIMS Results and Correlative Analysis Microscopy

**Figure 2:**
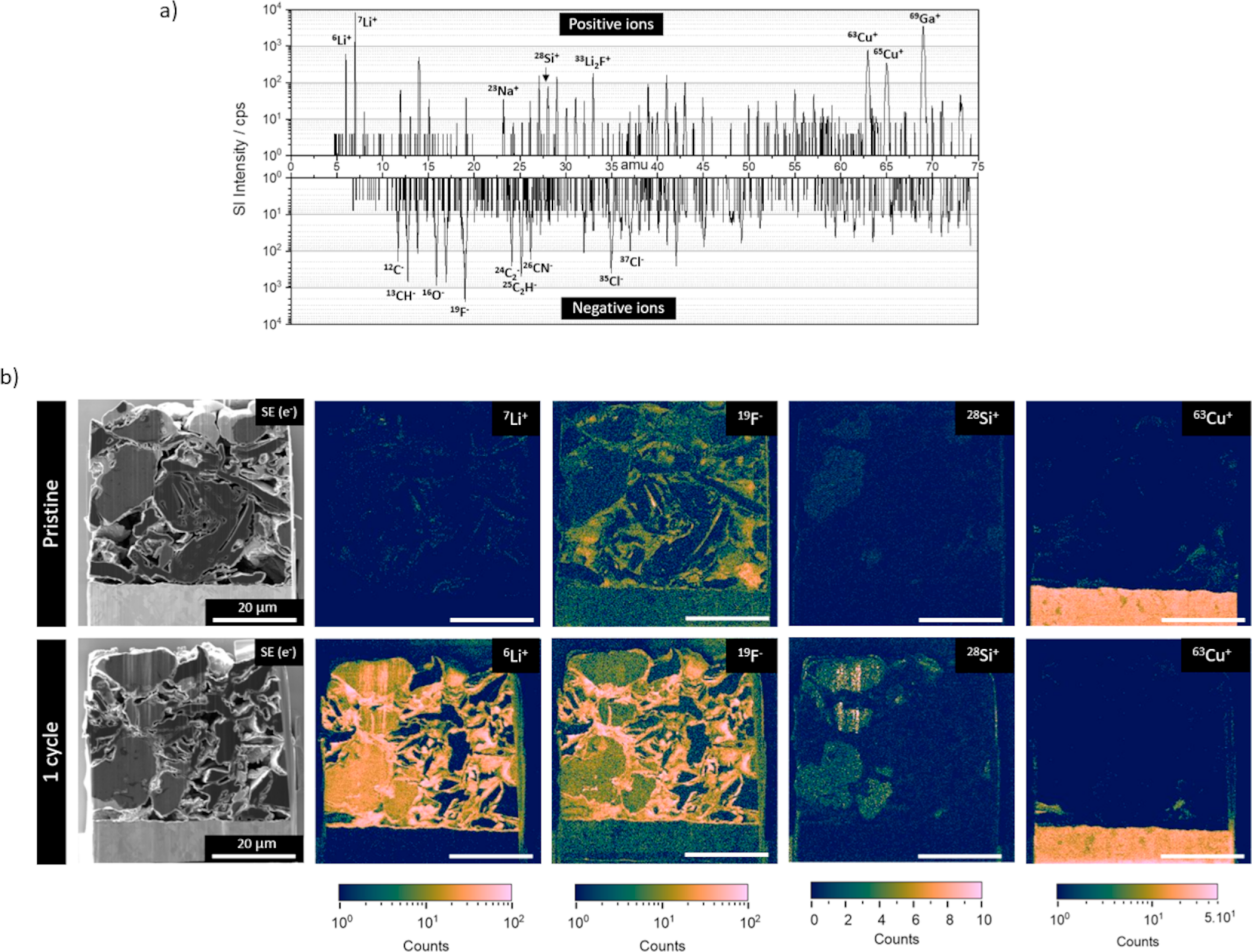
(a) MS of positive and negative SIs of the cycled anode with the most relevant peaks labeled. Total ion dose: 4.0 × 10^14^ ions/cm^2^ (0.64 pC/μm^2^). (b) SEM image of pristine (top row) and cycled (bottom row) lift-out specimens and SIMS images for ^6^Li, ^7^Li^+^, ^19^F^–^, ^28^Si^+^, and ^63^Cu^+^. All SIMS images obtained with 25 keV 2.5pA Ne^+^ primary ions. Images acquired in 512 × 512 pixels, with a dwell time of 2 ms/pixel (8 min of total acquisition). Ion dose 3.27 × 10^14^ ions/cm^2^ (0.52 pC/μm^2^). Images were binned to 256 × 256 pixels. Common scale bar of 20 μm.

The MS of the whole surface of the cycled electrode lift-out was obtained by FIB-SIMS (ZEISS NanoFab HIM equipped with LIST’s magnetic sector SIMS system, referred to as HIM-SIMS) to provide an overview of the surface chemical composition in both polarities (Figure 2a). The sample was transferred with argon-filled airtight shuttles between the preparation (Ga FIB-SEM-SIMS) and the analysis instrument (HIM-SIMS). The HIM-SIMS operated with a Ne^+^ primary ion beam was used for the SIMS analysis instead of the Ga-FIB-SEM-SIMS to avoid Ga implantation during SIMS analysis and to study the distribution of Ga implanted during the sample preparation step. MS is obtained using Ne^+^ primary ions by scanning the magnetic field from *B* = 140 to 300 mT, corresponding to a range mass of 2–93 amu. In positive mode, both lithium isotopes at 6 and 7 amu stand out at low masses with high intensity, while ^63^Cu and ^65^Cu from the current collector and implanted ^69^Ga dominate at high masses. The peak at 28 amu is associated with silicon and that at 33 amu is attributed to the Li_2_F^+^ cluster originating from the LiF compound, which constitutes an inorganic component of the SEI.^22−24^ A similar spectra fingerprint is obtained with the pristine sample (noncycled anode and without any contact with the electrolyte), except for the absence of lithium peaks and its cluster with fluorine Li_2_F^+^ (Figure S1). In negative mode, peaks are associated with carbon and its clusters (^13^CH^–^, ^14^CH_2_^–^, ^24^C_2_^–^, ^25^C_2_H^–^, and ^26^CN^–^), including the single hydrogenated ^13^CH^–^ and ^25^C_2_H^–^, which are more intense and preferred for imaging. Other relevant peaks correspond to oxygen clusters (^16^O^–^ and ^17^OH^–^) and fluorine at 19 amu. ^23^Na^+^, ^35^Cl^–^, and ^37^Cl^–^ are typical markers of environmental contamination, also perceptible in both MS.

SE images are recorded prior to SIMS images to provide morphologic and phase information for the same ROI (Figure 2b). The pixel dwell time, in the range of 1–2 ms/pixel, leads to a total acquisition time of 6–8 min for 512 × 512 pixels frame, underlining the high speed of these FIB-SIMS instruments in image mode. Four different SIs were selected (Li^+^, ^19^F^–^, ^28^Si^+^, and ^63^Cu^+^) and are summarized in Figure 2b for pristine and cycled samples. To avoid the saturation of the detectors (occurring at 5 × 10^5^ cps), the acquisition time or/and the primary current should be reduced; however, this also decreases the signal intensity for the other ions selected. In the case of lithium, which has a high ionization probability, thus high useful yields, the preferred option is to select the isotope with lower abundance when the content of Li is significant, i.e., ^6^Li instead of ^7^Li with 7.6% and 92.4% for natural abundance, respectively. The SIMS signal intensity per pixel can be increased by binning the image, and this can be useful for improved counting statistics, visualization, and interpretation. The copper signal originates at the current collector with traces in the free spaces between particles of the active material, presumably due to redeposition during ion polishing. The ^28^Si signal allows us to identify the silicon-rich particles, which are distinguished from graphite particles by BSE imaging (Figure S2). Fluorine and lithium are also recorded under similar analysis conditions for the pristine and cycled material. Although the pristine sample was not exposed to the fluorinated electrolyte and was not cycled, we detected both ^7^Li lithium and ^19^F fluorine. None of the raw materials for the manufacturing of the electrode have fluorine or lithium in their nominal composition. On the other hand, SIMS is very sensitive to fluorine and lithium due to their high electron affinity and high ionization potential, respectively. Thus, their detection in the pristine sample may only be due to the presence of traces of fluorine and lithium incorporated during the electrode manufacturing as the same machinery is also used for cathode materials containing lithium and typically bound with fluorinated compounds (e.g., polyvinylidene fluoride). In the cycled sample, ^19^F^–^ counts are higher due to the contribution of both the electrolyte decomposed compounds reduced during lithiation (i.e., growing of SEI) and the electrolyte residues (such as Li salts) remaining after the washing step. In the cycled sample, the lithium signal is much higher than in the pristine material, with a noticeable amount of lithium retained inside the particles (intercalated lithium or as a lithium alloy) and at the particle boundaries (presumably forming an SEI). Silicon–carbon particle interior poses high lithium signal, while graphitic particles show very low lithium signal. In silicon–carbon blended active materials, the delithiation first takes place in the graphite and proceeds to the silicon particles at a higher potential.^25^ Ex situ XRD spectra on the cycled electrode confirm the fully delithiated state of the graphite (results not shown here). This agrees with the higher lithium counting on silicon composite particles, in contrast with the very low signal on graphite particles. Conversely, lithium is observed in both silicon–carbon and graphite particles in the electrode in the lithiated state (Figure S3).

**Figure 3:**
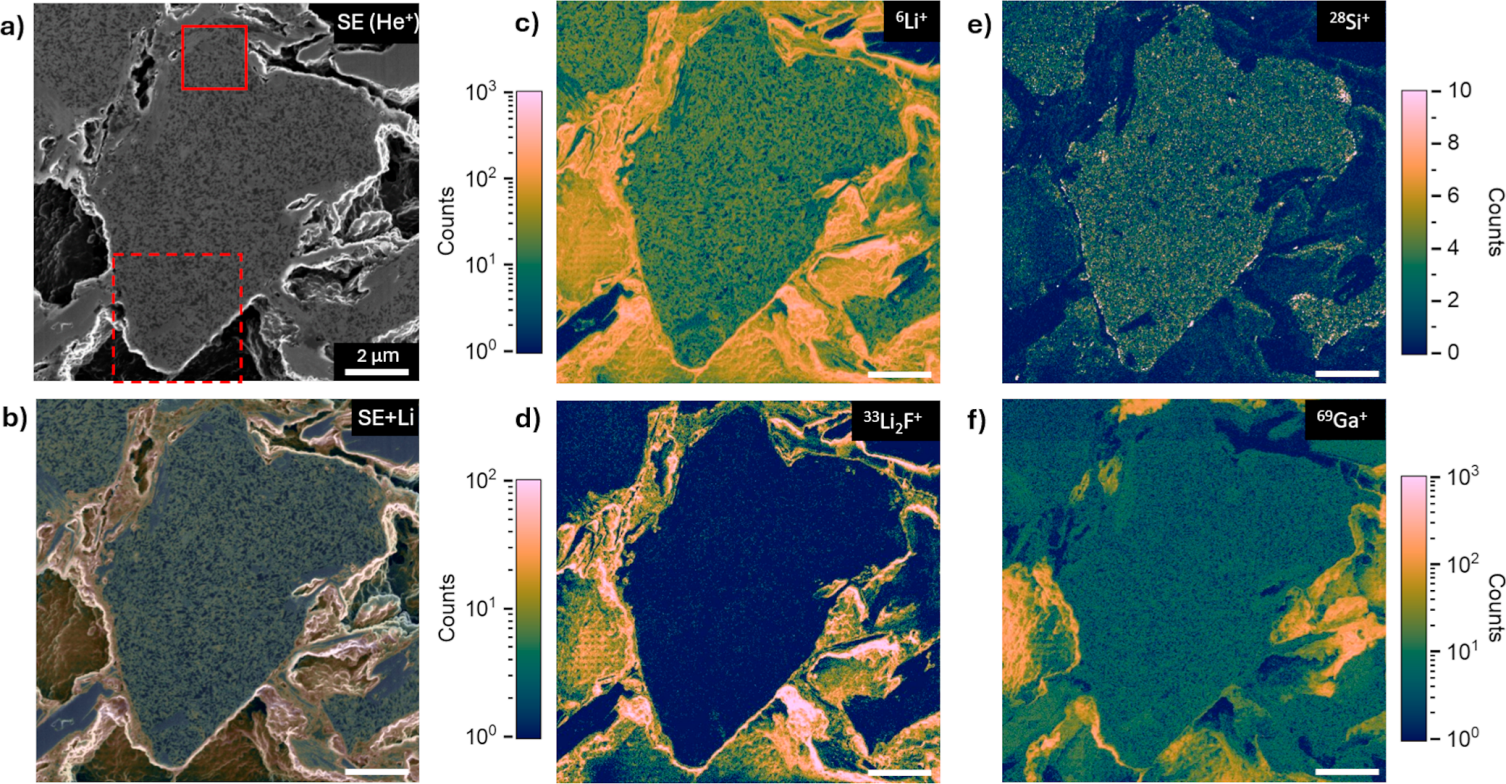
Correlative SE-SIMS analysis of a silicon–carbon particle of a cycled anode. Secondary electron image (a) and the corresponding ^6^Li, ^28^Si, ^33^Li_2_F, and ^69^Ga images (c–f). The correlative SE-SIMS image (b) for lithium is represented by overlay. Red continuous and dashed lines indicate regions of interest subsequently analyzed with smaller fields of view corresponding to Figures 4 and 5. SE image acquired with 25 keV 3pA He^+^ and 2048 × 2048 pixels. SIMS images obtained at 25 keV 5pA Ne^+^ and 1024 × 1024 pixels. Common scale bar of 2 μm.

SE and SIMS images, such as those in Figure 2b, provide a general overview of the structural and chemical information over the entire electrode thickness and, as a result, are useful for selecting a subregion of interest for further analysis. Note that the Ne^+^ ion dose accumulated after one acquisition in each positive and negative mode of 6.55 × 10^14^ ions/cm^2^ (1.04 pC/μm^2^) does not create any perceptible change of the roughness of the surface (Figure S4). Hence, the limited surface damage permits several SIMS image acquisitions without any intermediate repolishing step.^26,27^ In this way, successive analysis, focused on a single particle, was performed, as summarized in Figure 3. Secondary electron images, e.g., obtained with ion beams, combined with SIMS analytics information, provide to the best of our knowledge an unprecedented nanoscale correlative microscopy data set in terms of the high spatial resolution and high sensitivity. The enhanced SIMS signal originating in the particle boundaries and electrode pores can easily be included in the data interpretation thanks to the morphological information, and conversely, features detectable by SIMS can aid in identifying structural defects like cracks or small cavities hidden in the SE image (Figure S5). Moreover, these complementarity data, acquired in a single instrument, can be merged pixel-to-pixel to provide a correlative microscopy image, such as an overlay of the SE and SIMS (Figure 3b) to facilitate the data interpretation. It is worth noting that the combination of the field of view and raster size of the SIMS images in Figure 3 is appropriate for a complete survey of the surface under analysis as the radial extension of sputtered particles closely matches the image pixel size (23 × 23 nm^2^).^18^ The SI intensity is uniform from the core to the edge of the particles, as seen in Figure 3 and the line profiles in Figure S6. Furthermore, there is no SIMS shadowing effect despite the irregular surface shape outside the cross-section plane and the sharp particle edges. Material degradation, such as particle cracking, exfoliation, morphological and chemical changes at the particle surface, and film formation on particles, are typically aging mechanisms originating on the particle surface and expanding into the bulk of the particle upon evolution.^8^ Hence, the SI signal uniformity on the entire particle cross-section is valuable for consistent compositional comparisons from core to particle boundaries, which combined with SE-BSE images can give comprehensive chemical and structural information. The absence of shadowing effect by employing the lift-out method presented here is a remarkable difference with respect to the most used FIB cross-section workflow, which suffers from this artifact. Refer to Supporting Information for a more detailed comparison of these two methods. It is worth noting that the dot pattern (near the left center of Figure 3c–f) has been created during the tuning of the SIMS instrument (focalization, mass calibration, etc.) for this specific sample analyzed. This damage can be avoided by tuning the instrument in a different ROI.

**Figure 4:**
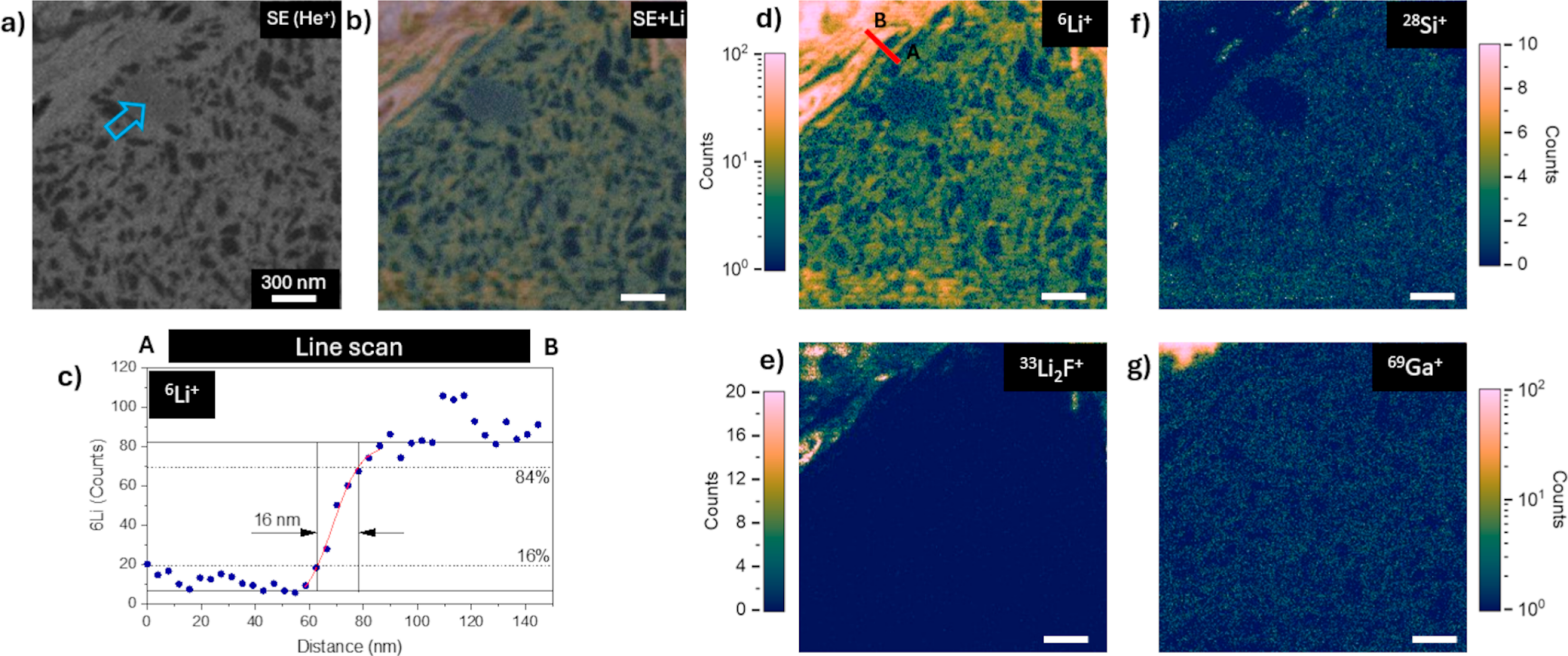
Evaluation of the SIMS lateral resolution. Scanned area of 2 × 2 μm^2^ of a silicon composite particle with SE (a), SIMS (d,f,e,g), and correlative SE with a ^6^Li SIMS image (b). A ^6^Li line scan with an intensity integrated over a width of 10 pixels, using a 16–84% maximum intensity variation criterion, results in a lateral resolution of 16 nm (c). SIMS acquisition was carried out with 512 × 512 pixels (3.9 nm/pixel), dwell time 1.5 ms/pixel, and 25 keV 2 pA Ne^+^ ion beam. Images are represented after binning, but the line scan is performed on the original data (512 × 512 pixels). The common scale bar is 300 nm.

The lateral resolution for SIMS imaging was evaluated on the cross-section of the active material particles. A subregion of a particle, marked with a box in a red continuous line in Figure 3, of 2 × 2 μm^2^, 512 × 512 pixels (3.9 nm/pixel) was analyzed with a 25 keV Ne^+^ 2 pA primary beam. These analytical conditions correspond with the optimal parameters to achieve a sub-15 nm lateral resolution (Figure 4c).^15^ A line scan on a ^6^Li image (over the original 512 × 512-pixel image), integrating the intensity over a width of 10 pixels and by implementing the 84–16% maximum intensity drop criterion, results in a lateral resolution of 16 nm, which corresponds to the expected range of the physical limit considering the collision cascade.^28^ Measurements elsewhere confirm this result (Figure S7). In Figure 4b is shown the correlative SE with the ^6^Li SIMS image. There is a clear correlation between the lithium intensity and the structure of the grain. The matrix is composed of nanosized silicon particles embedded in a carbon matrix with a graphitic material that is distinguished by its high energy density and electrode stability upon cycling.^16,29^ The silicon map allows the graphitic particles surrounded by the silicon–carbon compound (indicated with an arrow on the SE image in Figure 4a) and the grain boundary to be distinguished. The lithium map, with a higher signal dynamism than that of ^28^Si^+^, permits the nanostructure of the silicon–carbon compound to be resolved. Figure 4b indicates a higher Li count in the carbon matrix than in the silicon nanosized particles (identifiable by a rounded shape and with a lower SE emission than the carbon matrix). Moreover, the rise in lithium intensity in the left upper corner correlates with mass 33, probably corresponding to the inorganic LiF phase of SEI as ^7^Li_2_^19^F^+^ is located on the outer surface of the particle. The noticeable contrast between these two regions due to the lithium SI intensity is indicative of different types of materials. The difference in sputtering yield (density dependent), concentration of the species, chemical composition, and topography are the material-dependent factors that govern the SI signal intensity. The latter factor does not seem to be influencing the SI signal intensity, as shown in Figure 4, as the surface appears flat in the SE image. The densities of a growing phase obtained electrochemically (SEI) and the Si–C particle phase are probably not the same, which in turn impact the sputtering yield. Likewise, the lithium concentration in the SEI is different from that of the bulk of the particle. Finally, the different chemical composition of these two regions also affects the lithium signal intensity, with the presence of fluorine in the SEI probably playing an important role, thus increasing the ionization yield of positive ions, including lithium.^30,31^ Despite an optimal sample preparation, direct quantification cannot be performed, which is a weakness of the SIMS technique. However, it will allow isotopic measurements to be achieved across all the FIB lift-out specimens, e.g., ^6^Li/^7^Li for lithium isotopic tracing, in optimized analytical conditions. It should be mentioned that the gallium maps are very useful as indicators of the implanted and redeposited material during the sample preparation process; here, most of the image area has a low and homogeneous ^69^Ga signal indicative of low and uniform Ga implantation in the particle cross-section. Only a small section in the top left corner is noticeable due to its high intensity, indicating the boundary of the particle with the redeposited material of the Ga^+^ ion polishing in the last preparation step. Localized heating caused by the ion beam during sample preparation can induce artifacts. If thermal dissipation is poor, unacceptably high temperatures may develop, and thermal activation processes may occur (thereby increasing solid-state diffusivity and chemical reactivity). To overcome this damage, the use of cryo-FIB for sample preparation should be considered and compared to determine whether it has any effect on the results.^32^

**Figure 5:**
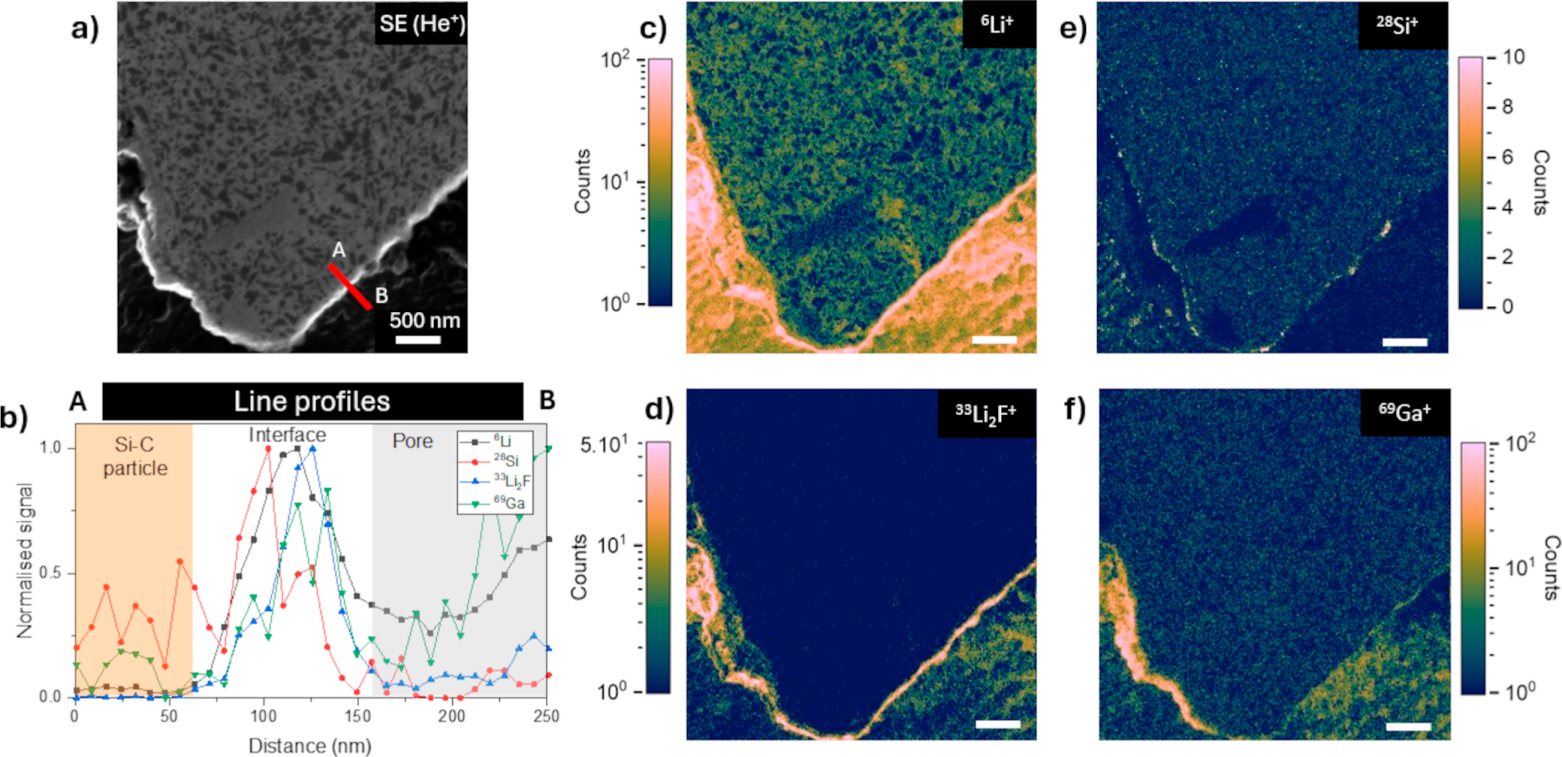
Particle interface analysis of the cycled anode. SE image (a), line profiles (b), and SIMS images of ^6^Li, ^28^Si, ^33^Li_2_F, and ^69^Ga (c–f) on the edge of the cross-sectioned silicon–carbon particle. Line profiles integrated over a width of 15 pixels (117 nm). The red arrow on the SE image indicates the location of the line profiles. The SIMS images are taken at 25 keV 1.5pA Ne^+^ 512 × 512 pixels, with a dwell time of 1.5 ms/pixel. The common scale bar is 500 nm.

The interfaces were analyzed under these analytical and sample preparation conditions. A new acquired image of the subregion in Figure 3 indicated by a dashed line and a line profile perpendicular to the particle edge is shown in Figure 5a. The smooth finishing on the particle cross-section and the sharp edge obtained by Ga-FIB machining facilitate SIMS data analysis from the bulk of the particle to its boundary. The detrimental effects of topography on the interpretation of SIMS data are thus strongly reduced. To exemplify this, in Figure 5b is shown the evolution of the normalized SI signals in the particle interface along a 117 nm-wide path (equivalent to 15 SIMS image pixels), with data points every 7.8 nm (equivalent to the pixel size). Lithium counts in the particle bulk are lower than those in the interparticle free space, which corresponds to the exterior surface of the particles laying underneath. A similar trend is observed for Li_2_F^+^, which has a negligible intensity in the bulk and a higher intensity in the particle exterior. Intercalated or alloyed lithium is detected in the bulk, whereas in the particle exterior, it may be due to the presence of LiF of the SEI and eventually electrolyte residues. The same analysis performed in an electrode in contact with the electrolyte for 1 h that has not been charged or discharged is shown in Figure S8. No SEI is expected to form on this electrode, and this is consistent with the very low signal for the full frame of the mass 33 amu or cluster Li_2_F^+^. In addition, the very low intensities of ^6^Li^+^ and Li_2_F^+^ indicate that the electrolyte residues on the electrode after washing are negligible. In Figure 5e, mass 28 is mainly detected in the bulk, and its signal intensity diminishes in the interparticle free space. A graphite particle, with no silicon in its nominal composition, is expected to be at the bottom of the pore. The interface is confined between the bulk and the interparticle free space or electrode porosity, and its extension is roughly indicated in the line scan plot (Figure 5b). The interface thickness, based on SIMS and SE images, is no more than 110 nm, therefore well in accordance with the literature for SEI formed at a low number of cycles.^33^ The peak intensity of ^6^Li and ^33^Li_2_F is indicative of a chemical variation, not only due to the formation of a passivation layer, i.e., SEI, but also due to an overall SI yield resulting from an edge effect (preferential sputtering on edges). Nevertheless, relative signal variations between different masses are of great interest as they are independent of the absolute SI intensity. The right-shifted peak position of Li_2_F^+^ with respect to that of ^28^Si supports the hypothesis of the formation of an LiF phase on the particle surface. Even with the sub-16 nm lateral resolution of this SIMS system, it is challenging to localize the SEI formed after 1 cycle, which is presumably not more than a few tens of nanometers thick but is most certainly identifiable for a longer cycling life with thicker SEI development and particle surface degradation.^34,35^ An equivalent methodology can be applied for studying the interfacial transition between a Si–C and graphite particle (Figure S9). It is worth noting that interface analysis with SIMS presents great difficulties if performed without preconditioning the electrode. Without the preparation method shown here, the roughness of the surface will result in uneven sputtering of the upper layers, creating topographical artifacts and hindering the interpretation of SIMS data. In addition, only the uppermost part of the electrode (electrode–electrolyte interface) can be accessed without our preparation methodology, while the interfaces at the depth cannot be investigated. Refer to Supporting Information for a fuller explanation.

The same sample preparation methodology and analysis have also proven to be effective for the SIMS imaging of cathode materials, such as NMC532 (Figure S10). SIMS images of the particle cross-section reveal nanosized domains, which are contrasted in ^6^Li and ^16^O by different signal intensities and in ^19^F and mass 33 amu images delimiting grain boundaries. NMC particles are composed of agglomerations of randomly oriented nanosized crystalline primary particles, thus giving the SIMS a crystallographic contrast. ^55^Mn and ^58^Ni maps show homogeneous distribution throughout the particle, and ^69^Ga is only detected on the outer grain surface. As the electrode is in pristine condition (never cycled and not exposed to electrolytes), the chemical composition is expected to be uniform and the structure undamaged. However, the combination of the cross-section and the SIMS sensitivity obtained is very useful to identify hotspots such as lithium trapping or depletion and determine size and its location with respect to the nanosized domains with a spatial resolution never before achieved.^36^

## 4 Conclusions

We presented a thorough workflow of specimen preparation for the chemical and morphological correlative image characterization of LIB electrodes using FIB-SIMS instruments. The methodology was applied to study the negative electrodes of liquid electrolyte half-cells under pristine conditions (without any contact with the electrolyte) and after one cycle in the delithiated state. The specimen preparation is carried out by using a novel FIB lift-out preparation protocol, allowing the site-specific extraction and study of the complete electrode thickness from the current collector to the electrode–electrolyte interface. Environmental contamination during the sample transfer from glovebox to the instrument is addressed by using airtight transfer systems. The complementarity of the chemical mapping and morphology data sets obtained by SIMS, ion-induced SE and SEM imaging, respectively, enables a cross-correlation of structural features and highly sensitive analytical information with an unprecedented high lateral resolution down to 16 nm. In these conditions, intraparticle domains, interfaces, and presumably the inorganic component of the SEI were observed. The combination of this methodology and FIB-SIMS capability represents a breakthrough compared to previous studies for a highly sensitive, high spatial resolution correlative analysis of battery electrode materials and promises to be of great use in other applications, such as multilayer structures and coatings.

## Data Availability

The data that support the findings of this study are available from the corresponding authors on reasonable request.

## Supporting Information Available

Additional characterization of pristine and cycled sample; SIs line profiles of the silicon–carbon composite and graphite particles and their interfaces; SE and SIs images of NMC532 lift-out; SIMS data assessment, and comparison with conventional SIMS analysis strategies (PDF)

## References

[ref1] Goodenough J. B.; Kim Y. Challenges for rechargeable Li batteries. Chem. Mater. 2010, 22 (3), 587–603. 10.1021/cm901452z.

[ref2] Tarascon J.-M.; Armand M. Issues and challenges facing rechargeable lithium batteries. Nature 2001, 414 (6861), 359–367. 10.1038/35104644. 11713543

[ref3] Palacín M. R. Understanding ageing in Li-ion batteries: A chemical issue. Chem. Soc. Rev. 2018, 47 (13), 4924–4933. 10.1039/C7CS00889A. 29745954

[ref4] Kabir M. M.; Demirocak D. E. Degradation mechanisms in Li-ion batteries: a state-of-the-art review. Int. J. Energy Res. 2017, 41 (14), 1963–1986. 10.1002/er.3762.

[ref5] Hervig R. L.; Mazdab F. K.; Williams P.; Guan Y.; Huss G. R.; Leshin L. A. Useful ion yields for Cameca IMS 3f and 6f SIMS: Limits on quantitative analysis. Chem. Geol. 2006, 227 (1–2), 83–99. 10.1016/j.chemgeo.2005.09.008.

[ref6] Berthault M.; Buzlukov A.; Dubois L.; Bayle P. A.; Porcher W.; Gutel T.; De Vito E.; Bardet M. Lithium isotope tracing in silicon-based electrodes using solid-state MAS NMR: a powerful comprehensive tool for the characterization of lithium batteries. Phys. Chem. Chem. Phys. 2023, 25 (33), 22145–22154. 10.1039/D3CP02646A. 37563981

[ref7] Berthault M.; Santos-Pena J.; Lemordant D.; Vito E. D. Dynamics of the 6Li/7Li Exchange at a Graphite-Solid Electrolyte Interphase: A Time of Flight-Secondary Ion Mass Spectrometry Study. J. Phys. Chem. C 2021, 125 (11), 6026–6033. 10.1021/acs.jpcc.0c10398.

[ref8] Waldmann T.; Iturrondobeitia A.; Kasper M.; Ghanbari N.; Aguesse F.; Bekaert E.; Daniel L.; Genies S.; Gordon I. J.; Löble M. W.; et al. Review—Post-Mortem Analysis of Aged Lithium-Ion Batteries: Disassembly Methodology and Physico-Chemical Analysis Techniques. J. Electrochem. Soc. 2016, 163 (10), A2149–A2164. 10.1149/2.1211609jes.

[ref9] Lombardo T.; Walther F.; Kern C.; Moryson Y.; Weintraut T.; Henss A.; Rohnke M. ToF-SIMS in battery research: Advantages, limitations, and best practices. J. Vac. Sci. Technol., A 2023, 41 (5), 053207 10.1116/6.0002850.

[ref10] ChiuHuang C. K.; Zhou C.; Shadow Huang H. Y. In situ imaging of lithium-ion batteries via the secondary ion mass spectrometry. J. Nanotechnol. Eng. Med. 2014, 5 (2), 021002 10.1115/1.4028010.

[ref11] Zhou Y.; Su M.; Yu X.; Zhang Y.; Wang J. G.; Ren X.; Cao R.; Xu W.; Baer D. R.; Du Y.; et al. Real-time mass spectrometric characterization of the solid–electrolyte interphase of a lithium-ion battery. Nat. Nanotechnol. 2020, 15 (3), 224–230. 10.1038/s41565-019-0618-4. 31988500

[ref12] Sector Field Mass Spectrometry for Elemental and Isotopic Analysis; Prohaska T., Irrgeher J., Zitek A., Jakubowski N., Eds.; Royal Society of Chemistry, 2014.

[ref13] Pillatsch L.; Östlund F.; Michler J. FIBSIMS: A review of secondary ion mass spectrometry for analytical dual beam focussed ion beam instruments. Prog. Cryst. Growth Charact. Mater. 2019, 65 (1), 1–19. 10.1016/j.pcrysgrow.2018.10.001.

[ref14] De Castro O.; Audinot J. N.; Hoang H. Q.; Coulbary C.; Bouton O.; Barrahma R.; Ost A.; Stoffels C.; Jiao C.; Dutka M.; et al. Magnetic Sector Secondary Ion Mass Spectrometry on FIB-SEM Instruments for Nanoscale Chemical Imaging. Anal. Chem. 2022, 94 (30), 10754–10763. 10.1021/acs.analchem.2c01410. 35862487 PMC9352148

[ref15] Audinot J. N.; Philipp P.; De Castro O.; Biesemeier A.; Hoang Q. H.; Wirtz T. Highest resolution chemical imaging based on secondary ion mass spectrometry performed on the helium ion microscope. Rep. Prog. Phys. 2021, 84 (10), 105901 10.1088/1361-6633/ac1e32. 34404033

[ref16] Bridel J.-Sébastien. Umicore Progress in Si-Anode Materials: To Be the First European Silicon Anode Player at Scale. In Advanced Automotive Battery Conference AABC, Mainz, 2023.

[ref17] Wu J.; Ihsan-Ul-Haq M.; Chen Y.; Kim J. K. Understanding solid electrolyte interphases: Advanced characterization techniques and theoretical simulations. Nano Energy 2021, 89, 106489 10.1016/j.nanoen.2021.106489.

[ref18] Wirtz T.; Philipp P.; Audinot J. N.; Dowsett D.; Eswara S. High-resolution high-sensitivity elemental imaging by secondary ion mass spectrometry: From traditional 2D and 3D imaging to correlative microscopy. Nanotechnology 2015, 26 (43), 434001 10.1088/0957-4484/26/43/434001. 26436905

[ref19] De Castro O.; Biesemeier A.; Serralta E.; Bouton O.; Barrahma R.; Hoang Q. H.; Cambier S.; Taubitz T.; Klingner N.; Hlawacek G.; et al. npSCOPE: A New Multimodal Instrument for in Situ Correlative Analysis of Nanoparticles. Anal. Chem. 2021, 93 (43), 14417–14424. 10.1021/acs.analchem.1c02337. 34670088

[ref20] Crameri F.; Shephard G. E.; Heron P. J. The misuse of colour in science communication. Nat. Commun. 2020, 11 (1), 5444 10.1038/s41467-020-19160-7. 33116149 PMC7595127

[ref21] Volkert C. A.; Minor A. M. Focused Ion Beam Microscopy and Micromachining. MRS Bull. 2007, 32 (5), 389–399. 10.1557/mrs2007.62.

[ref22] Cox X. B.; Linton R. W.; Bursey M. M. Formation of small cluster ions from alkali halides in SIMS, 1984, 55( (3), ), 281, 10.1016/0168-1176(84)87091-3

[ref23] Liu Z.; Lu P.; Zhang Q.; Xiao X.; Qi Y.; Chen L. Q. A Bottom-Up Formation Mechanism of Solid Electrolyte Interphase Revealed by Isotope-Assisted Time-of-Flight Secondary Ion Mass Spectrometry. J. Phys. Chem. Lett. 2018, 9 (18), 5508–5514. 10.1021/acs.jpclett.8b02350. 30198721

[ref24] Estel J.; Hoinkes H.; Kaarmann H.; Nahr H.; Wilsch H. On the problem of water adsorption on alkali halide cleavage planes, investigated by secondary ion mass spectroscopy. Surf. Sci. 1976, 54, 393–418. 10.1016/0039-6028(76)90233-8.

[ref25] Yao K. P. C.; Okasinski J. S.; Kalaga K.; Almer J. D.; Abraham D. P. Operando Quantification of (De)Lithiation Behavior of Silicon–Graphite Blended Electrodes for Lithium-Ion Batteries. Adv. Energy Mater. 2019, 9, 1803380 10.1002/aenm.201803380.

[ref26] Bordes A.; De Vito E.; Haon C.; Boulineau A.; Montani A.; Marcus P. Multiscale Investigation of Silicon Anode Li Insertion Mechanisms by Time-of-Flight Secondary Ion Mass Spectrometer Imaging Performed on an in Situ Focused Ion Beam Cross Section. Chem. Mater. 2016, 28 (5), 1566–1573. 10.1021/acs.chemmater.6b00155.

[ref27] Budnik G.; Scott J. A.; Jiao C.; Maazouz M.; Gledhill G.; Fu L.; Tan H. H.; Toth M. Nanoscale 3D tomography by in-flight fluorescence spectroscopy of atoms sputtered by a focused ion beam. Nano Lett. 2022, 22 (20), 8287–8293. 10.1021/acs.nanolett.2c03101. 36215134

[ref28] Wirtz T.; Vanhove N.; Pillatsch L.; Dowsett D.; Sijbrandij S.; Notte J. Towards secondary ion mass spectrometry on the helium ion microscope: An experimental and simulation based feasibility study with He + and Ne + bombardment. Appl. Phys. Lett. 2012, 101, 041601 10.1063/1.4739240.

[ref29] Wu J.; Cao Y.; Zhao H.; Mao J.; Guo Z. The critical role of carbon in marrying silicon and graphite anodes for high-energy lithium-ion batteries. Carbon Energy 2019, 1 (1), 57–76. 10.1002/cey2.2.

[ref30] Pillatsch L.; Wirtz T. SIMS using O -F -Cl -Br - and I - primary ion bombardment. Surf. Interface Anal. 2012, 44 (10), 1370–1372. 10.1002/sia.5066.

[ref31] Priebe A.; Pethö L.; Michler J. Fluorine Gas Coinjection as a Solution for Enhancing Spatial Resolution of Time-of-Flight Secondary Ion Mass Spectrometry and Separating Mass Interference. Anal. Chem. 2020, 92 (2), 2121–2129. 10.1021/acs.analchem.9b04647. 31858788

[ref32] Zachman M. J.; Tu Z.; Archer L. A.; Kourkoutis L. F. Nanoscale Elemental Mapping of Intact Solid-Liquid Interfaces and Reactive Materials in Energy Devices Enabled by Cryo-FIB/SEM. ACS Energy Lett. 2020, 5 (4), 1224–1232. 10.1021/acsenergylett.0c00202.

[ref33] Peled E.; Menkin S. Review—SEI: Past, Present and Future. J. Electrochem. Soc. 2017, 164 (7), A1703–A1719. 10.1149/2.1441707jes.

[ref34] Dupré N.; Moreau P.; De Vito E.; Quazuguel L.; Boniface M.; Bordes A.; Rudisch C.; Bayle-Guillemaud P.; Guyomard D. Multiprobe Study of the Solid Electrolyte Interphase on Silicon-Based Electrodes in Full-Cell Configuration. Chem. Mater. 2016, 28 (8), 2557–2572. 10.1021/acs.chemmater.5b04461. 27212791 PMC4869615

[ref35] Veith G. M.; Doucet M.; Baldwin J. K.; Sacci R. L.; Fears T. M.; Wang Y.; Browning J. F. Direct Determination of Solid-Electrolyte Interphase Thickness and Composition as a Function of State of Charge on a Silicon Anode. J. Phys. Chem. C 2015, 119 (35), 20339–20349. 10.1021/acs.jpcc.5b06817.

[ref36] Sui T.; Song B.; Dluhos J.; Lu L.; Korsunsky A. M. Nanoscale chemical mapping of Li-ion battery cathode material by FIB-SEM and TOF-SIMS multi-modal microscopy. Nano Energy 2015, 17, 254–260. 10.1016/j.nanoen.2015.08.013.

