# Supplementary material for: Lift-Out Specimen Preparation and Multiscale Correlative Investigation of Li-Ion Battery Electrodes Using Focused Ion Beam-Secondary Ion Mass Spectrometry Platforms
Source: ACS Appl Mater Interfaces. 2024 Oct 9;16(42):57141–50. doi: 10.1021/acsami.4c12915 (PMC11503607; doi:10.1021/acsami.4c12915)
Supplement: Supplementary file 1 — am4c12915_si_001.pdf [file am4c12915_si_001.pdf]

Supporting Information:

Lift-out specimen preparation and multiscale correlative investigation of Li-ion battery electrodes using Focused Ion Beam-Secondary Ion Mass Spectrometry platforms

Pablo Maria Delfino[1,2,*], Mariia Bofanova[3,4], Eric De Vito[3], Nicolas Dupré[4], Guillaume Lamblin[5], Willy Porcher[3], Tom Wirtz[1] and Jean-Nicolas Audinot[1]

1. Advanced Instrumentation for Nano-Analytics (AINA), Luxembourg Institute of Science and Technology (LIST), L-4422 Belvaux, Luxembourg

2. University of Luxembourg, L-4365 Esch-sur-Alzette, Luxembourg

3. University of Grenoble Alpes, CEA-Liten, 38000 Grenoble, France

4. Nantes Université, CNRS, Institut des Matériaux de Nantes Jean Rouxel, IMN, 44000 Nantes, France

5. Transparent and Optically Tuneable Materials and Nanostructures, Luxembourg Institute of Science and Technology (LIST), L-4422 Belvaux, Luxembourg

*Corresponding author: pablo-maria.delfino@list.lu

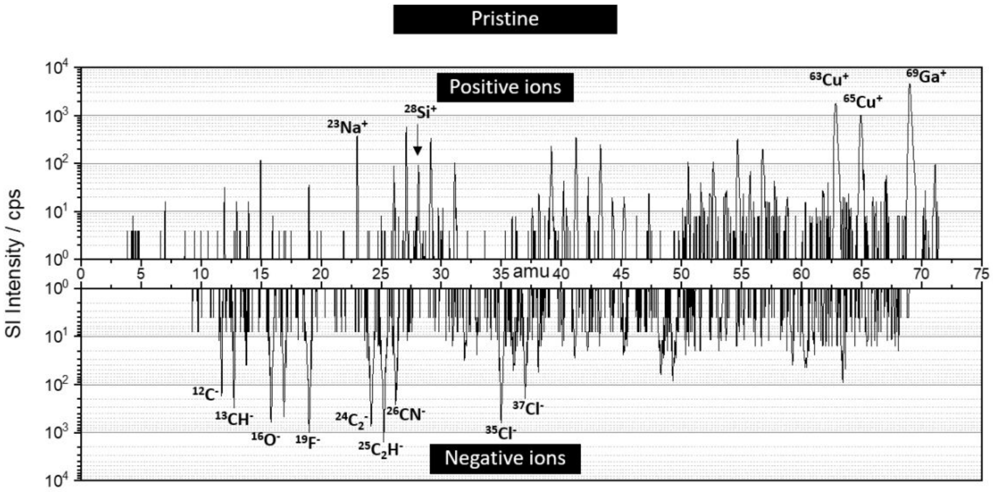

Figure S1. Mass spectrum of positive and negative secondary ions of a pristine sample. 25 keV 3 pA Ne^+ primary ions. Total ion dose of 4.0 x 10^{14} ions/cm^2 (0.64 pC/μm^2).

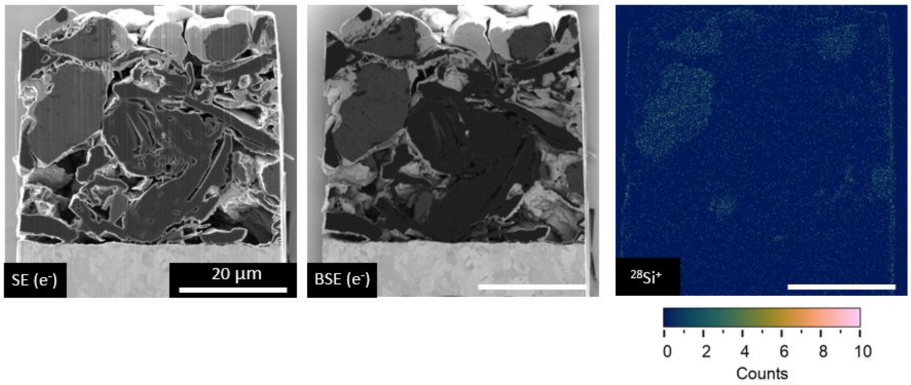

Figure S2. Contrast between a silicon compound and graphite particles in different image modes: secondary electron SE, backscattered electron BSE, and Si mapping with SIMS. Electron images taken at 5 keV and 1.6nA.

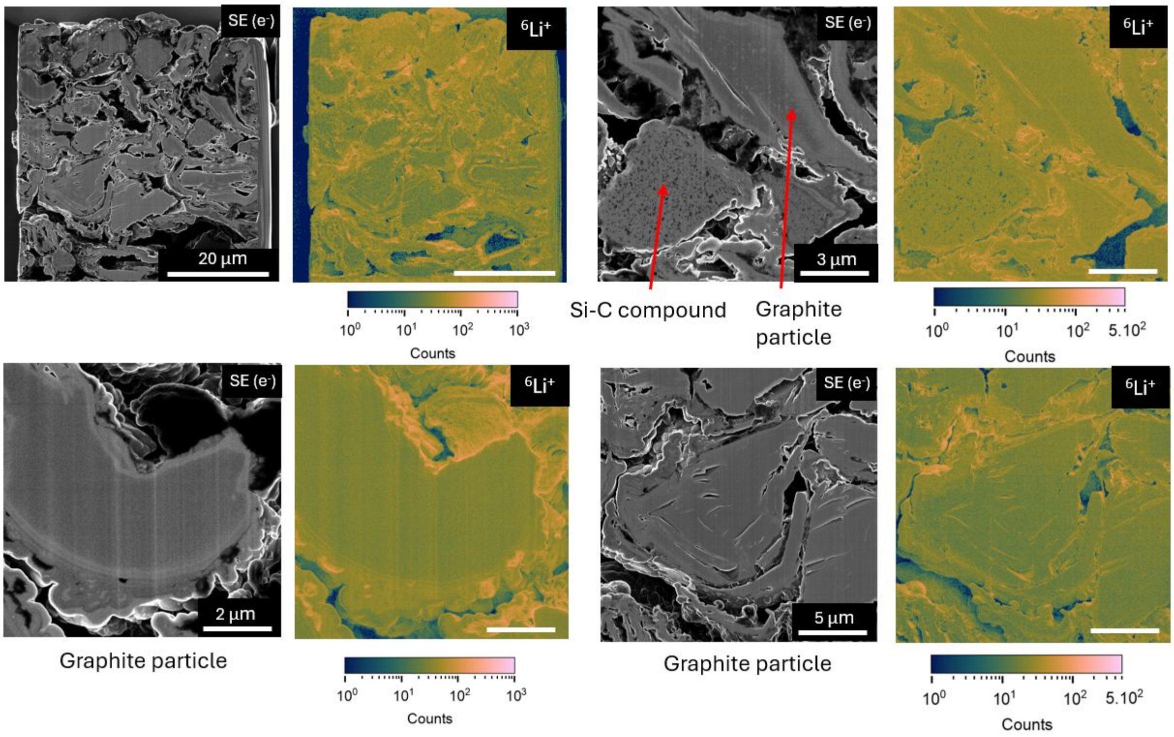

Fig. S3. Analysis of a lithiated negative electrode. SE images and 6Li+ maps by SIMS for different regions of interest. SIMS images obtained with 25keV 2pA Ne^+ primary ions. Images acquired in 1024 x 1024 pixels, dwell time of 1.5 ms/pixel (26 min of acquisition time).

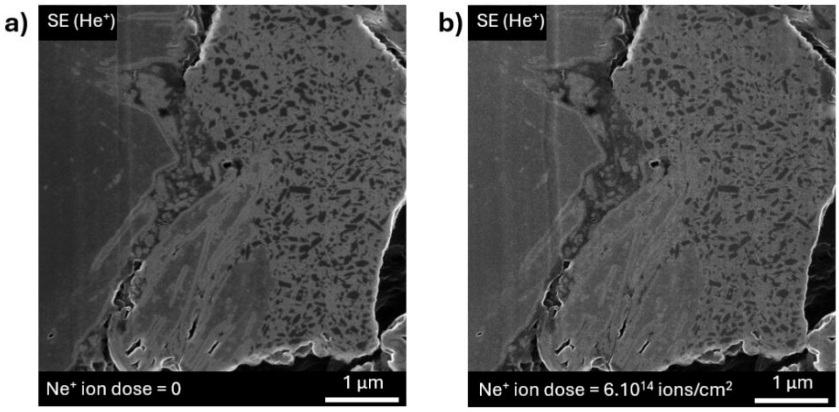

Figure S4. Secondary electron images of pristine electrode before (a) and after (b) SIMS analysis with a final 25 keV Ne^+ dose of 6.10^{14} ions/cm^2. No noticeable change in roughness is observed.

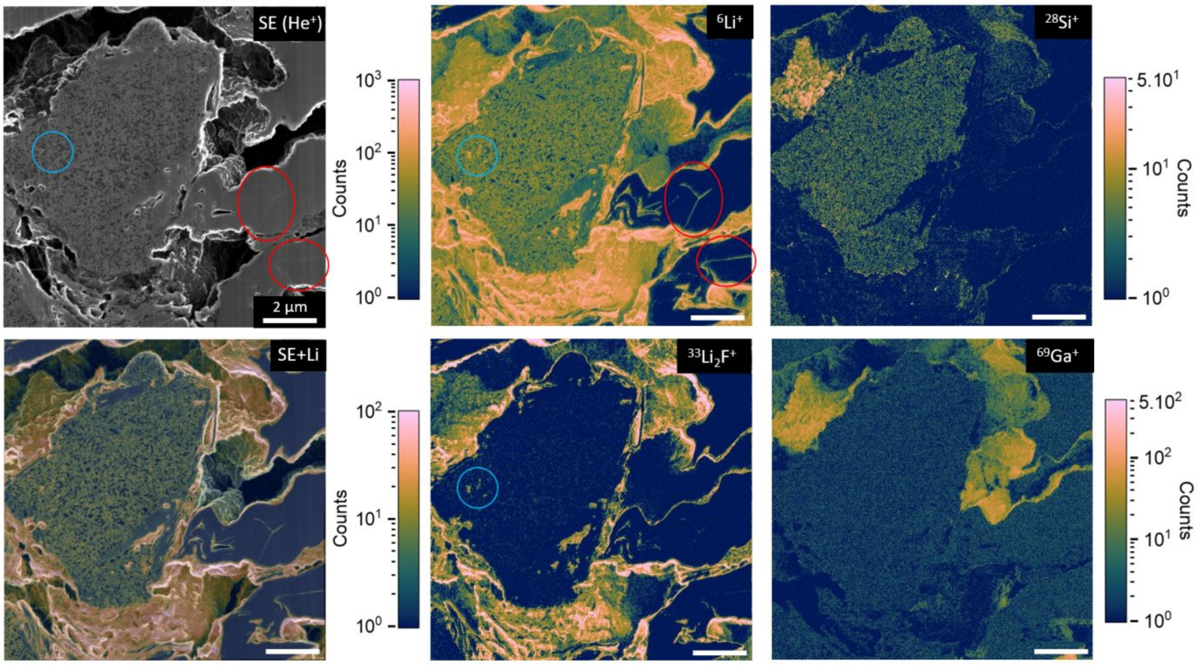

Figure S5. Correlative SE-SIMS helps find morphological features not evident in SE images. Here, cracks on graphite particles (red ovals) and voids (light blue circle) are easily found with Li^+ and Li_2F^+ SIMS imaging. SE-HIM 25keV 3pA He^+. SIMS images 25 kV 5pA Ne^+ 1024x1024 binned to 512x512 pixels, 1.5 ms of dwell time. Ion dose of 3.4×10^{15} ions/cm^2 (54.6 pC/µm^2). Common scale bar of 2 µm.

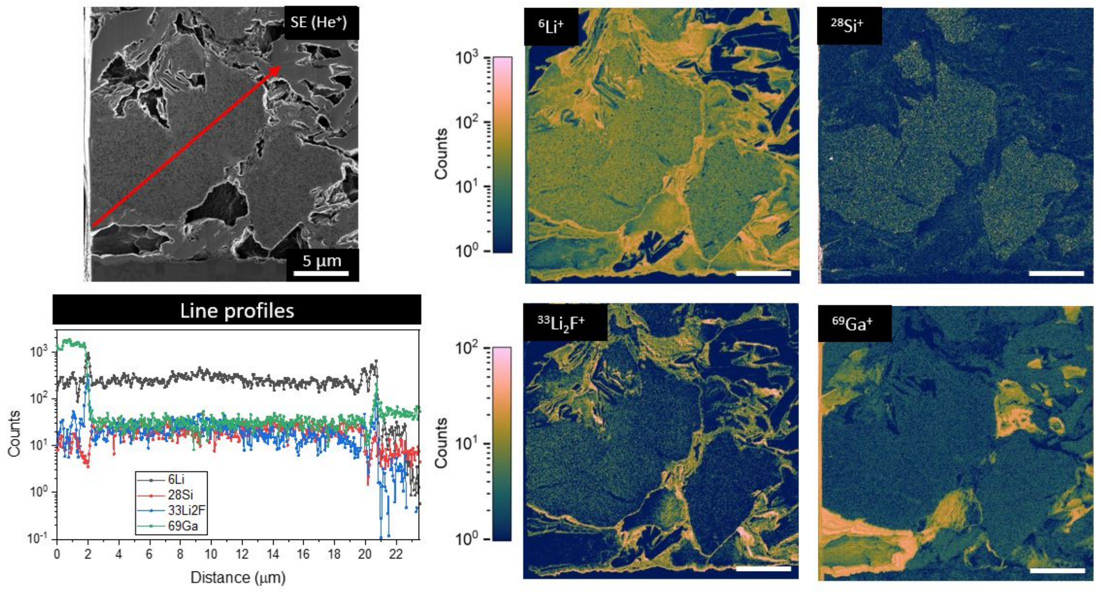

Figure S6. Line profile over a large silicon composite particle showing uniform SI signal intensities within the particle. The signal is integrated over a line width of 10 pixels, equivalent to 0.49 µm. SIMS images 25 kV 5pA Ne^+ 1024x1024 binned to 512x512 pixels, 1.5 ms of dwell time. Ion dose of 7.9×10^{14} ions/cm^2 (12.6 pC/µm^2). Common scale bar of 5 µm.

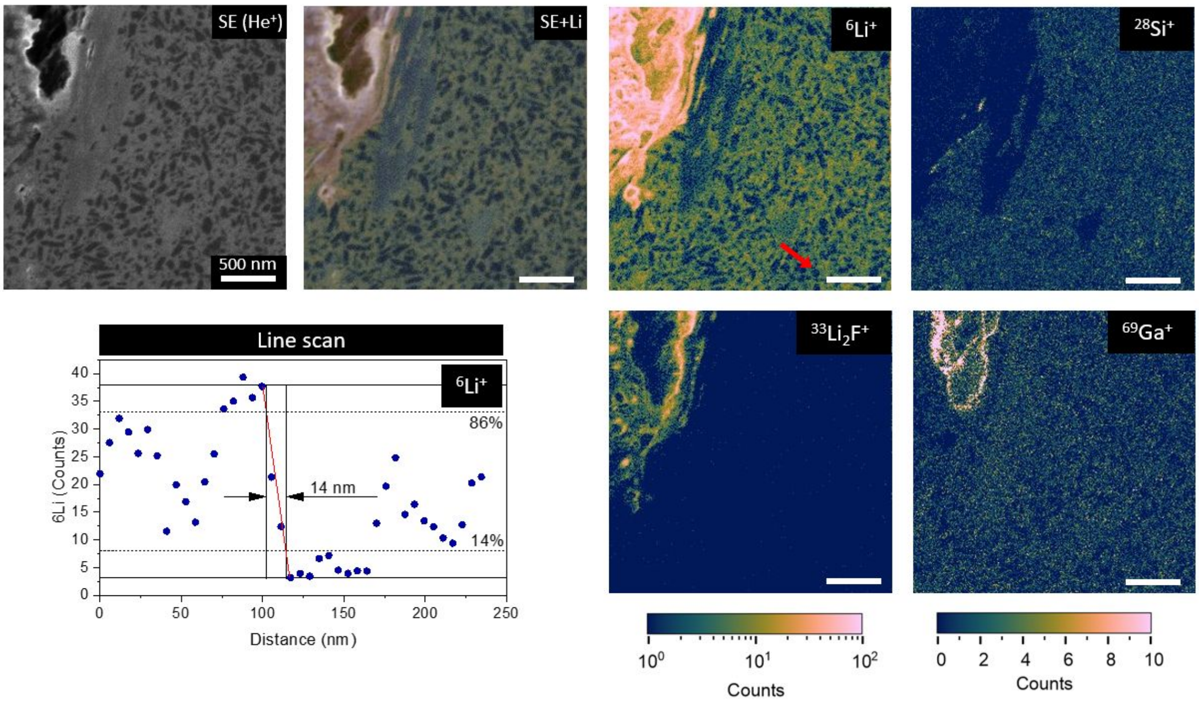

Figure S7. Evaluation of the SIMS lateral resolution in a different ROI to that shown in Figure 3. A scanned area of 3x3 μm^2 of a silicon composite particle with SE, SIMS, and correlative SE with a ^{6}Li SIMS image. A ^{6}Li line scan with a 15-pixel integrated intensity and using a 84-16% maximum intensity drop criterion results in a lateral resolution of 14 nm. SIMS acquisition was taken with 512x512 pixels (5.9 nm/pixel), a dwell time of 1.5 ms/pixel and a 25 keV 2 pA Ne^+ ion beam. The common scale bar of 500 nm.

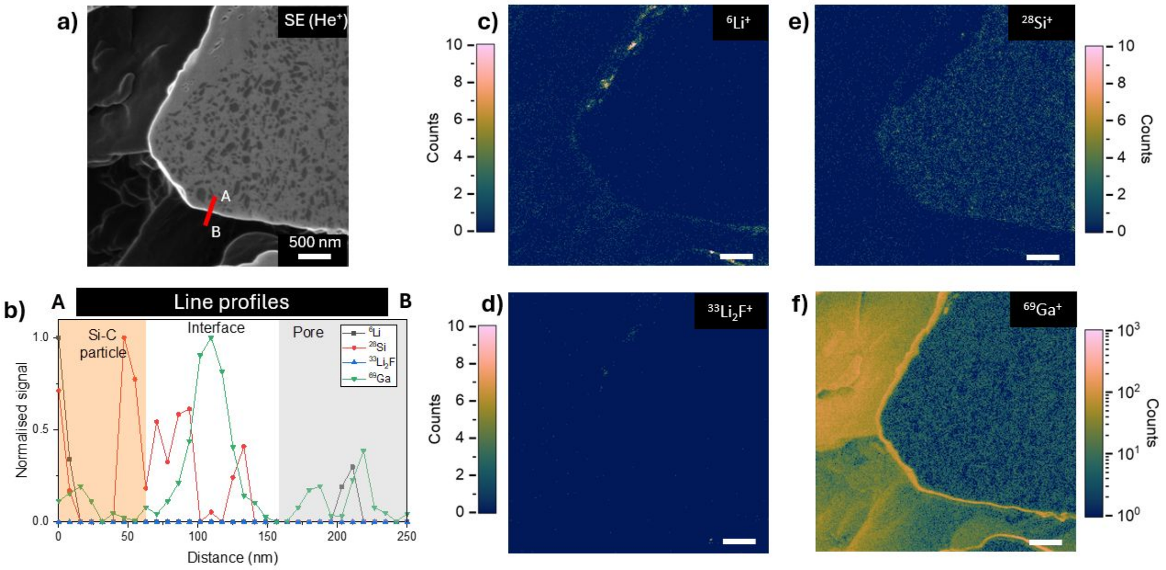

Figure S8. Particle interface analysis of an anode exposed to electrolyte for 1h. Line profiles and SIMS images of ^{6}Li, ^{28}Si, ^{33}Li$_2$F, and ^{69}Ga on the edge of the cross-sectioned silicon-carbon particle. Line profiles integrated over a width of 15 pixels (117 nm). The red line on the SE image indicates the location of the line profiles. The SIMS images are taken at 25 keV 1.5pA Ne^{+} 512 x 512 pixels, with a dwell time of 1.5 ms/pixel. The common scale bar is 500 nm.

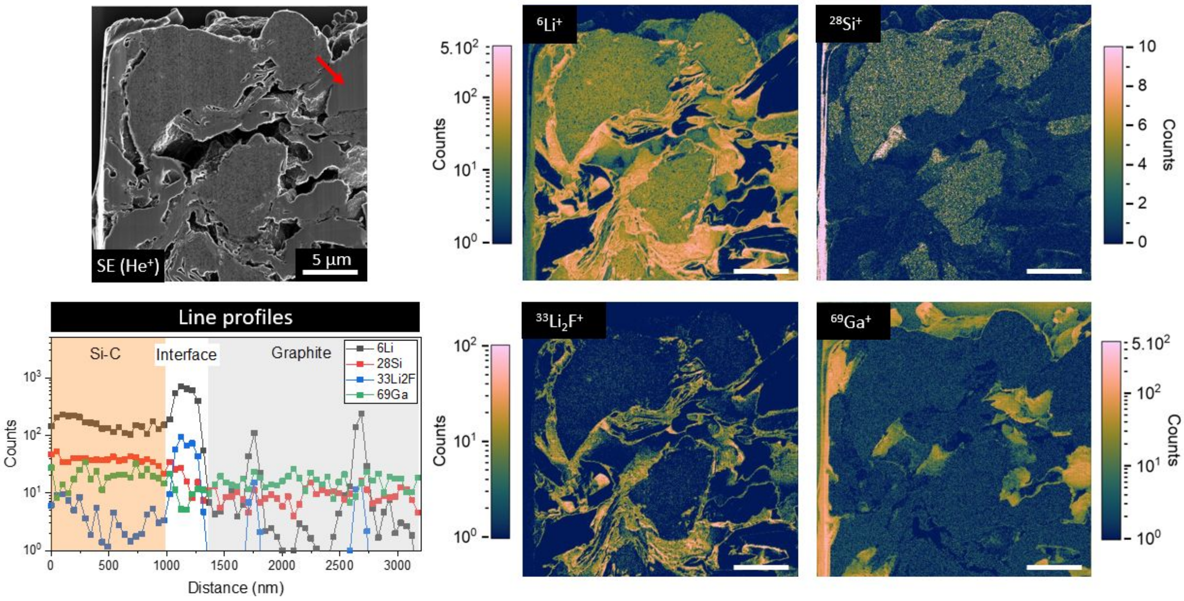

Figure S9. Interface study between an Si-C and graphite particle. The line profiles with a 10-pixel (490 nm) integrated intensity on cross-sectioned particles, indicated by the arrow on the SE image. Distinctive signal intensities facilitate the identification of the two different particles and the interface between them. SIMS images 25 keV 5pA Ne^{+} 1024x1024 pixels, dwell time of 1.5 ms/pixel. The common scale bar of 5 µm.

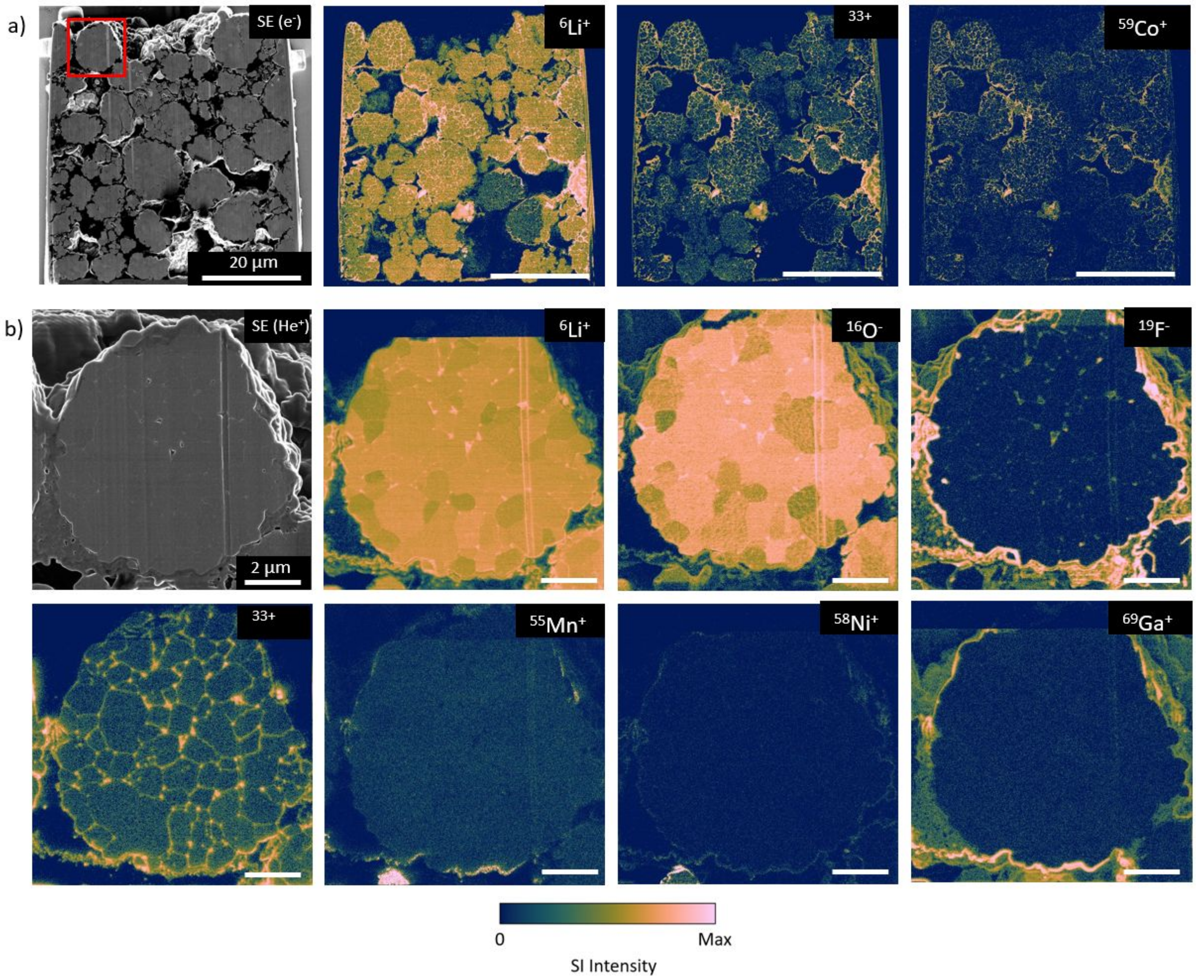

Figure S10. SE images of a pristine NMC532 cathode material lift-out specimen next to a selection of SIMS images surveying: (a) the entire electrode thickness. Scale bar of 20 µm; and (b) a single particle. The scale bar of 2 µm. A 10 nm Au coating before SIMS analysis improves the secondary ion yield. 25keV Ne^{+}, 5-10 pA, 512x512 and 1024x1024 pixels, dwell time of 1.5-2 ms/pixel.

SIMS data assessment

On unprepared battery electrode, a prominent roughness can be observed, as seen in Figure S11a with the electrode-electrolyte interface of a pristine anode. Sequential SIMS image acquisitions, so-

called 3D imaging, to monitor the 2D chemical composition in depth, tend to exacerbate this effect. This approach cannot be subjected to real 3D reconstructions as these consider the surface flat and the analyzed volume a cuboid, and, consequently, cannot provide elemental and isotopic distribution through the electrode without interpretation artifacts. Figure S11b shows a sequential SIMS image taken on the uppermost surface of the pristine anode at 30 keV Ga^+ and 10 pA beam current (conditions necessary to achieve a SIMS spatial resolution better than 50 nm) [1]. The non-uniform erosion created by the variations of the sputtering rate in the 3 directions induced typical tapered shape artifacts (Figure S11 c-d), thus not reflecting the original morphology. Under these conditions, the analysis is limited to a depth of only 1 µm for 14 h acquisition time (Figure S11 e).

A conventional FIB cross-section was also considered as a simpler and cheaper methodology capable of achieving equivalent surface quality for SIMS image analysis. Figure S12a depicts the SE image of the plane N revealing the subsurface material structure, which in this case makes it possible to study the entire electrode thickness, that is, the active material from the top surface to the current collector. However, in both positive and negative modes, the ion detection is non-uniform due to the shadowing effect on the inclined surface, as shown in Figure S12b. Gallium is implanted in the region both after the ion milling process and inherently during SIMS analysis. The line profiles 2 on the current collector with counts dropping from 10^3 to a few counts next to the left and right flank, respectively. The same trend is noticed on the line profile 4 for carbon signal. This artifact originates from the distortion of the extraction field due to abrupt shape irregularities, forcing the SI to follow trajectories off to the secondary ion optical axis with lower transmission, thereby inducing local underdetection, namely a shadow effect [2,3]. This artifact was not observed for samples prepared with the lift-out method, as shown in Figure S13 and S14.

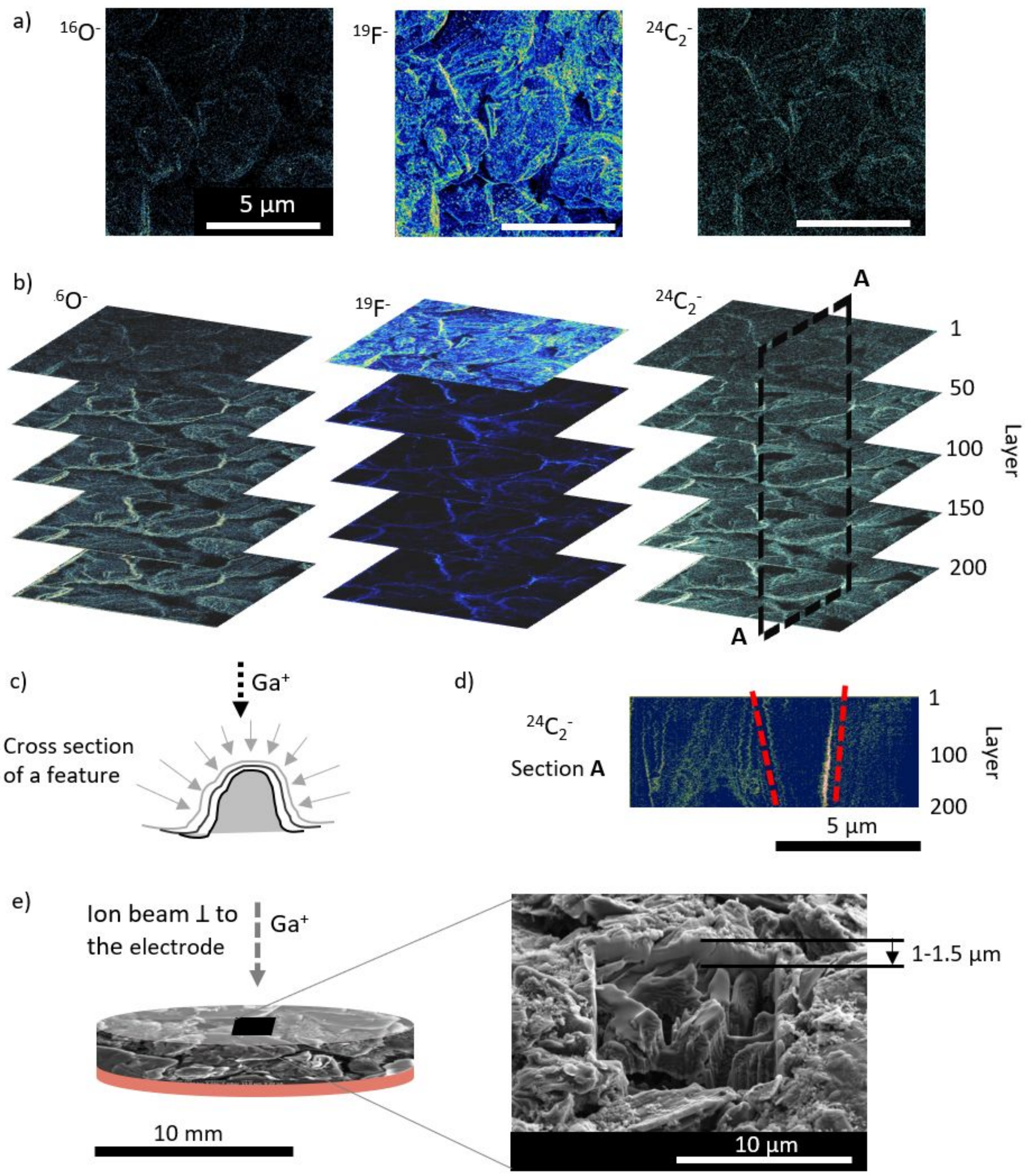

Figure S11. 2D and 2D sequential SIMS imaging of a pristine anode. (a) $^{16}O^-$, $^{19}F^-$ and $^{24}C_2^-$ images of the first layer; (b) image stack of negative ions $^{16}O^-$, $^{19}F^-$ and $^{24}C_2^-$ of the selected layer are shown here; (c) Schema of the shape evolution of a surface feature by the progressive material remotion due to differential sputtering rates and (d) cone-like artifacts induced in 3D stack assemblies; (e) SE-SEM image of the crater after SIMS analysis. SIMS images were taken with 10 pA (Ga^+) at 30keV, an image raster of 512 x 512 pixels and a dwell time of 1 ms. The FoV is 10 µm. Hence, the dose per image is 1.64×10^{16} ions/cm^2 (26.2 pC/µm^2).

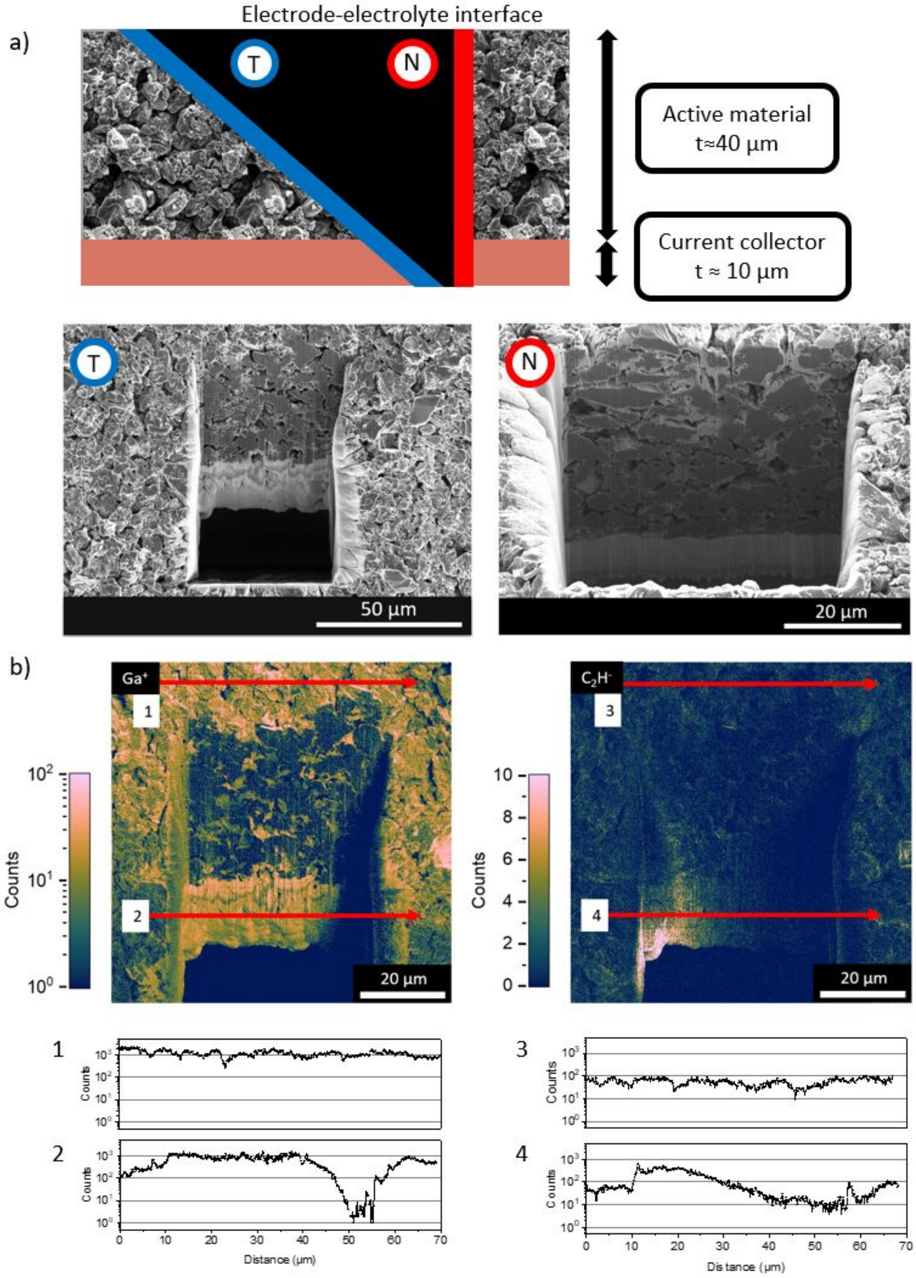

Figure S12. Cross-section SIMS analysis scheme. (a) Ga FIB milling of a triangular shaped trench with access to the entire electrode thickness, including active material and current collector. SE-SEM images of normal N and tilted T planes. (b) SIMS images of Ga^+ and C_2H^- with a shadow effect. Line profiles 1 and 3 on regions outside the milled area show uniform ion detection; profiles 2 and 3 through the trench bottom exhibit an abrupt drop on the right flank. Images of 512 x 512 pixels, 1.5

ms of dwell time, and 30 keV Ga^+ ion beam with currents of 50 pA and 10 pA for positive and negative mode were used, respectively.

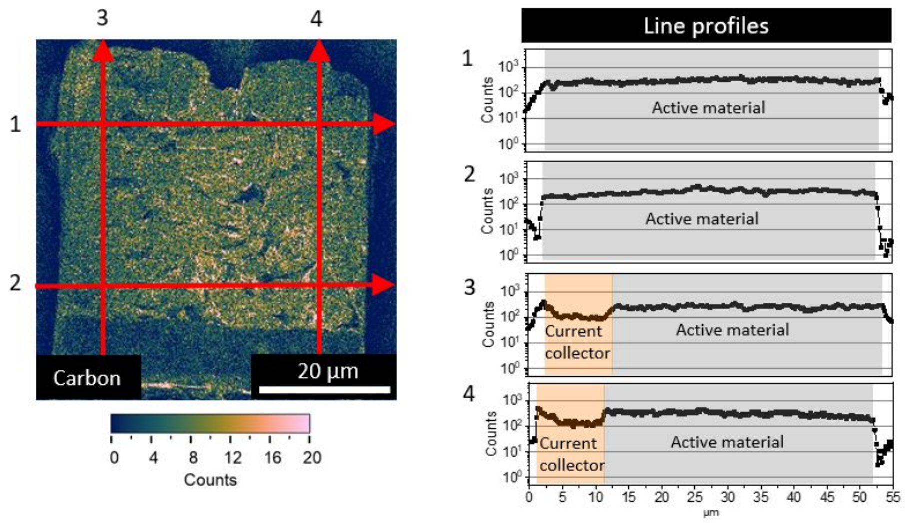

Figure S13. SIMS carbon image of a lift-out specimen of pristine anode. Line profiles show the uniformity of the signal detection through horizontal and vertical directions. 25keV 3pA Ne^+ primary ions. Image raster 512x512 binned to 256x256 pixels, 2 ms of pixel dwell time, thus leading to a dose of 3.25 x 10^{14} ions/cm^2 (0.52 pC/µm^2).

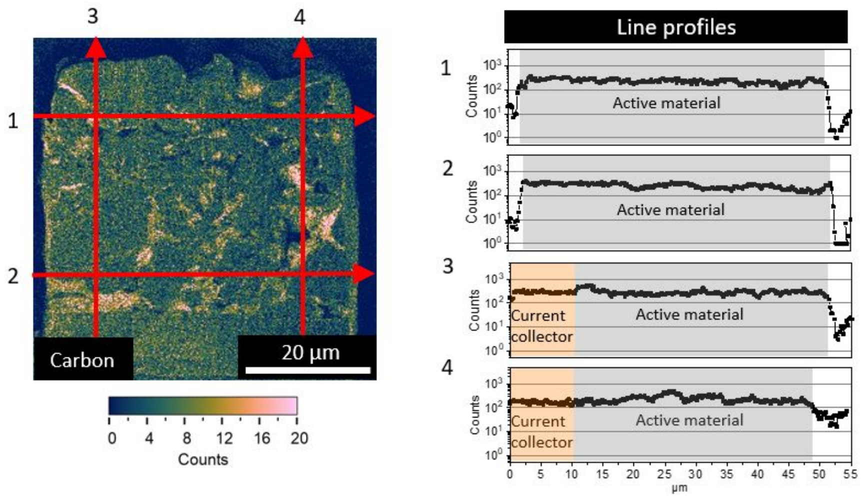

Figure S14. SIMS carbon image of a cycled sample after lift-out specimen preparation. Line profiles show the uniformity of the signal detection in horizontal and vertical directions. 25keV 3pA Ne^+ primary ions. Image raster 512x512 binned to 256x256 pixels, dwell time of 2 ms/pixel, thus leading to a dose 3.25×10^{14} ions/cm^2 (0.52 pC/µm^2).

References

1. De Castro, O. *et al.* Magnetic Sector Secondary Ion Mass Spectrometry on FIB-SEM Instruments for Nanoscale Chemical Imaging. *Anal Chem* **94**, 10754–10763 (2022).
2. Lee, J. L. S., Gilmore, I. S., Fletcher, I. W. & Seah, M. P. Topography and field effects in the quantitative analysis of conductive surfaces using ToF-SIMS. *Appl Surf Sci* **255**, 1560–1563 (2008).
3. Rangarajan, S. & Tyler, B. J. Topography in secondary ion mass spectroscopy images. *Journal of Vacuum Science & Technology A: Vacuum, Surfaces, and Films* **24**, 1730–1736 (2006).
